# Supplementary material for: Utility of the Age Discrepancy between Frailty-Based Biological Age and Expected Life Age in Patients with Urological Cancers
Source: Cancers (Basel). 2022 Dec 17;14(24):6229. doi: 10.3390/cancers14246229 (PMC9776733; doi:10.3390/cancers14246229)
Supplement: Supplementary file 1 [file cancers-14-06229-s001.zip › Supplementary Files.pdf]

Togazhi K., et al

Supplementary Files

Table S1. background of participants

	Non-cancer individuals
Number	1790
Age, years (IQR)	53 (38-66)
Male, n	730 (41%)
Body mass index, kg/m^2 (IQR)	23 (20-25)
Handgrip, kg	28 (23-37)
Timed get up and go (TGUG), sec.	4.9 (4.3-5.7)
Fatigue (yes), n	233 (13%)
Depression (yes), n	122 (6.8%)
Hypertension, n	542 (30%)
Diabetes mellitus, n	123 (6.9%)
Cardiovascular disease, n	118 (6.6%)
Albumin, g/dL	4.4 (4.3-4.6)
Hemoglobin, g/dL	14 (13-15)
eGFR, ml/min/1.73m^2 (IQR)	82 (71-93)
Frailty discriminant score (FDS)	–0.63 (–1.05 - –0.14)

IQR: interquartile range

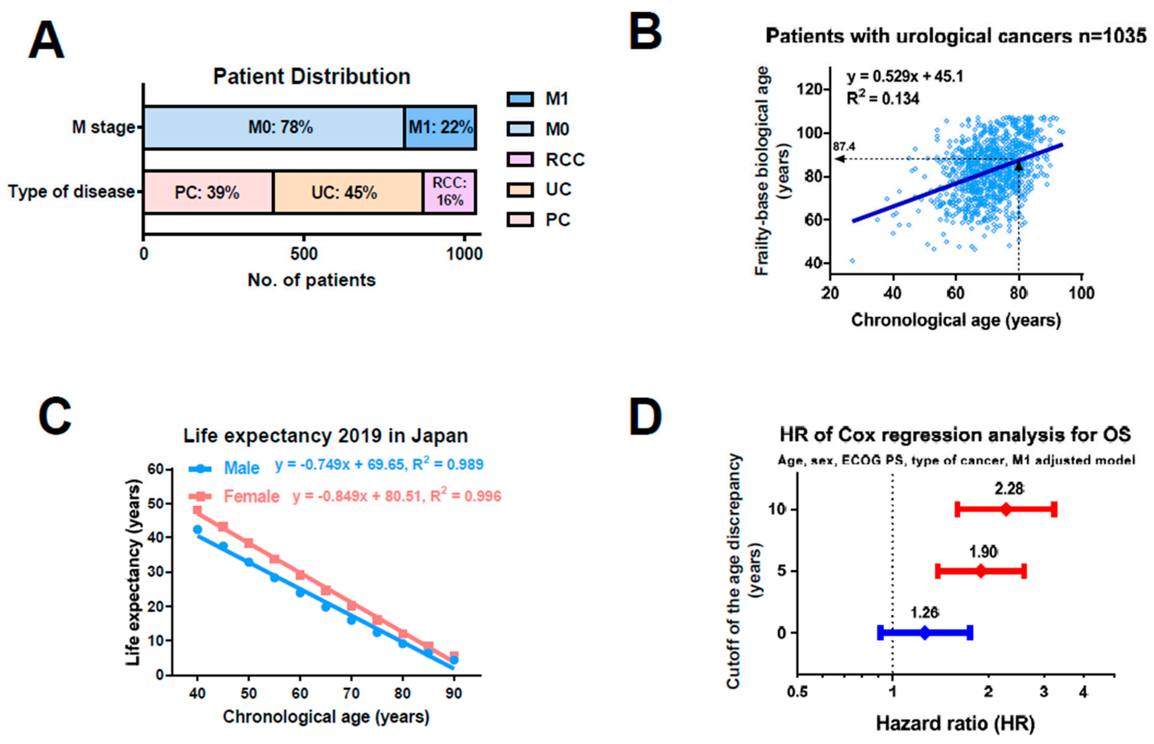

Figure S1. Distribution of Patients, association of ages, the life expectancy 2019 in Japan, and the effect of the cutoff of the age discrepancy on overall survival

A: Distribution of Patients with urological cancers. **B:** Association between the frailty-based biological age and chronological age. **C:** Life expectancy 2019 in Japan. **D:** The effect of the cutoff of the age discrepancy on overall survival.

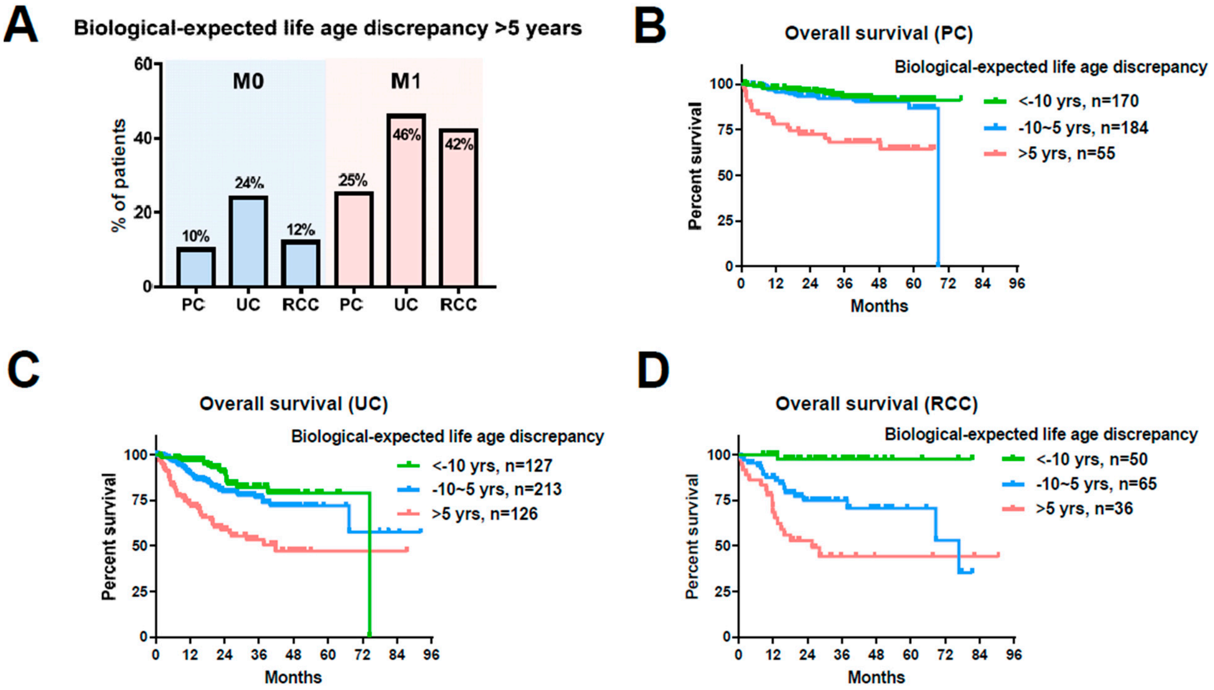

Figure S2. The prevalence of patients with the biological-expected life age discrepancy of >5 years and the effect of biological-expected life age discrepancy on the overall survival

A: The prevalence of patients with the biological-expected life age discrepancy of >5 years. **B:** The effect of biological-expected life age discrepancy on the overall survival of patients with PC. **C:** The effect of biological-expected life age discrepancy on the overall survival of patients with UC. **D:** The effect of biological-expected life age discrepancy on the overall survival of patients with RCC.

Supplemental Excel: FDS calculator

FDS calculator for patients with non-PC and PC.
